# Comparison of the RSNA chest CT classification system and CO-RADS system in reporting COVID-19 pneumonia in symptomatic and asymptomatic patients

**DOI:** 10.1186/s43055-022-00798-w

**Published:** 2022-05-24

**Authors:** Aliaa S. Sheha, Nada H. Mohamed, Yara M. Eid, Dina S. Sheha, Mohamed El-Shayeb, Mariam M. Amin, Alia Mohammed Saeed, Dina Abdou, Ahmed M. Osman

**Affiliations:** 1 Department of Diagnostic & Interventional Radiology and Molecular Imaging, Faculty of Medicine - Ain Shams University, Cairo, Egypt; 2 Internal Medicine, Endocrinology and Metabolism Department, Faculty of Medicine - Ain Shams University, Cairo, Egypt; 3 Internal Medicine, Allergy and Clinical Immunology Department, Faculty of Medicine - Ain Shams University, Cairo, Egypt; 4 Internal Medicine and Hematology Department, Faculty of Medicine - Ain Shams University, Cairo, Egypt

**Keywords:** Coronavirus, COVID-19, Computed tomography (CT), CO-RADS, RSNA

## Abstract

**Background:**

Coronavirus disease (COVID-19) is a new infection with three pandemic waves up till now. CT plays an important role in diagnosis with multiple reporting systems that can be used during CT analysis. We aimed to compare reporting using the recommendations of the radiological society of North America (RSNA) versus the coronavirus disease reporting and data system (CO-RADS) and to assess the performance of CT if used in asymptomatic patients as a screening. Two hundred and fifty-one patients who underwent chest CT scanning either due to clinical suspicion or as screening before hospital admission were included in this retrospective observational cross-sectional study. This was followed by RT-PCR for confirmation. Three radiologists with different years of experience interpreted the CT findings using the RSNA recommendations and the CO-RADS reporting. The data were collected and compared.

**Results:**

There was no statistically significant difference noted in the diagnostic accuracy obtained while using the RSNA recommendations and the CO-RADS reporting system. Also, a good inter-rater agreement was noticed while using the two reporting systems. The CT showed a highly significant value while used in the assessment of symptomatic patients in controversy to the screening of asymptomatic patients.

**Conclusion:**

Both reporting systems show similar diagnostic accuracy with a good almost similar inter-rater agreement. Both can be used while interpreting the CT images of cases with suspected COVID-19 infection. CT can be used effectively in the detection of COVID-19 infection between symptomatic patients while it is of a lower value in the screening of asymptomatic patients.

## Background

Coronavirus disease 2019 (COVID-19) infection was first reported in December 2019 in Wuhan, China [1]. The widespread human-to-human transmission led to the declaration of a pandemic by the World Health Organization (WHO) on January 30, 2020 [2].

Although the gold standard for diagnosis of COVID-19 is the reverse transcription-polymerase chain reaction (RT-PCR) assay, its sensitivity ranges from 42 to 83% depending on the viral load, in addition, there is a relative scarcity in some developing countries that occurred during the first wave and the long time it takes for the results to be delivered [3]. With the COVID-19 pandemic, many rapid tests appeared depending on rapid detection of the IgG and IgM within 10 to 30 min providing a rapid decision yet still of much lower diagnostic accuracy compared to the RT-PCR test [4].

This led to the evolution of the role of computed tomography (CT) in many countries as a screening tool for patients with clinically suspected COVID-19 pneumonia [5]. COVID-19 has CT-specific features yet may show some overlap with other diseases especially viral pneumonia [6]

In an attempt to standardize CT reporting in patients with suspected COVID-19, the Radiological Society of North America (RSNA) Expert Consensus Statement was published [7]. In early March 2020, the Dutch Radiological Society developed a standardized reporting system for reporting suspected cases of COVID-19 pneumonia, which was named COVID-19 Reporting and Data System (CO-RADS) [5]. Reporting cases requires the presence of high interobserver agreement to avoid confusion and guide physicians during patient management [8]. This was the hypothesis of this article trying to show the degree of interobserver agreement between different reporting systems that are commonly used during CT interpretation of cases with suspected COVID-19 infection to avoid clinicians’ confusion while using any of them. To our knowledge, limited studies are available comparing the two reporting systems and correlating them to RT-PCR findings, and showing the difference in CT accuracy while used in the screening of asymptomatic patients.

We aimed to compare the current reporting systems for suspected cases of COVID-19 pneumonia (RSNA reporting system and CO-RADS classification) and to correlate them with RT-PCR results. Also, we assessed the degree of interobserver agreement in both reporting systems.

The secondary aim was to assess the performance of CT in the screening of asymptomatic patients.

## Methods

### Patients

Two hundred and fifty-one patients were referred from the Emergency Room (ER) or the Triage clinic of Ain Shams University Hospital (a tertiary hospital that was allocated to partially receive COVID-19 cases) to the Radiology Department for a non-contrast CT study of the chest to exclude the presence of COVID-19 infection, followed by RT-PCR that had been taken within the same day. They were enrolled in this retrospective observational cross-sectional study. The study was conducted over the period from June 2020–May 2021. The authors received the ethical approval to use the patients’ data and images in the Picture Archiving and Communication System (PACS) from the Faculty of Medicine - Ain Shams Unniversity Ethical Committee, and the patients’ consent was waived being a retrospective study.

### Inclusion criteria

Any patient with clinical suspicion of COVID-19 infection, and any asymptomatic patients who were indicated for hospital admission or prepared for any surgical interference who underwent non-contrast CT to screen for COVID-19 infection, followed by RT-PCR.

### Exclusion criteria

Patients with non-available RT-PCR results and patients with excessive breathing motion artifacts that affected the accuracy of the CT image interpretation.

### Patients’ data

The patients’ records were revised to collect their demographic data including age and sex. Also, the presence of any comorbidities was recorded (Diabetes mellitus DM, hypertension HTN, cardiac disease, chronic kidney disease, chronic liver disease, and chronic respiratory disease). The patients presenting symptoms were recorded from their records to classify them into cases having symptoms of COVID-19 infection and asymptomatic cases. Symptomatic cases were defined as those with any of the following symptoms (Fever, cough, dyspnea, sore throat, malaise, myalgia, anosmia, ageusia, and gastrointestinal symptoms including anorexia, nausea, and diarrhea,) [9]

### CT Technique

- The study was performed using the Prime Aquilion 80-slice CT machine (Toshiba, US). The patients were placed in a supine position with their arms elevated above their heads. All patients were instructed to hold their breaths with full inspiration for as long as they could, to minimize the breathing motion artifacts.
- CT parameters included tube voltage of 120 kV, mA ranging between 150 and 400 mA according to the patient’s weight, 1.25 mm slice thickness, 0.625 mm slice interval, 512 × 512 matrix, and tube speed 35 mm/rotation (0.5 s rotation time). In pediatric cases, a low -dose CT technique was used via reducing the Kv and mA, and in some pediatric cases with the motion not controlled, oral sedation was used under the supervision of an anesthesiologist.
- Image processing and interpretation: The images were transferred to the workstation (Fuji Synapse workstation or Paxera-Ultima workstation) for image interpretation by three independent readers. (One radiologist with 14 years of experience, the second with 10 years of experience, while the third was a fellow with only 5 years experience in chest radiology.) All radiologists depend on their observation and experience without using any special algorithms or deep learning systems to measure the inter-rater agreement. Each reader was blinded to the results of RT-PCR, the clinical condition of the patients apart from being a suspicious case of COVID-19 infection, and the interpretation of the other radiologists.
- The following radiological points were recorded to estimate the COVID-19 infection probability:
  - Presence of ground-glass opacity (GGO) with or without septal thickening, crazy paving pattern, consolidation, and fibrosis.
  - The distribution of the GGO within the lung was either peripheral or central, unilateral or bilateral, and uni-lobar or multi-lobar.
  - The presence of a reverse halo sign.
  - The presence of other CT features of pneumonia as lobar consolidation, pulmonary cavity/abscess, and pulmonary nodules with a tree in bud appearance.
- Each CT scan was evaluated according to the RSNA recommendations and CO-RADS reporting system as follows:
  - Reporting the case according to the RSNA recommendations into the typical, indeterminate, atypical, or negative probability for COVID-19 infection [7].
  - Reporting the case according to CO-RADS into CO-RADS 1–5 with CO-RADS 1 was considered negative, while CO-RADS 5 was considered a very high probability case [5].
  - The presence of bilateral multi-lobar predominantly peripheral patchy areas of GGO was considered the most typical form of COVID-19 infection consistent with CO-RADS 5.

### RT-PCR assessment

Nasopharyngeal or oropharyngeal swab was done for each patient on the same day of the CT study. The RT-PCR tests were repeated twice only for the cases with negative swabs and persistent clinical suspicion.

### Sample size calculation

Using PASS program version 11 for sample size calculation (NCSS, LSS Statistical Software, USA), setting power at 80% and alpha error at 0.05, according to the previous literature the expected AUC for diagnostic validity of CO-RADS for diagnosis of COVID-19 pneumonia is about 0.80, assuming a difference of 0.10 in AUC between CO-RADS and RSNA reporting system for diagnosis of COVID-19 pneumonia, a sample size of at least 150 suspected patients were needed (75 cases with positive RT-PCR and 75 cases with negative RT-PCR).

### Statistical methods

Data were analyzed using IBM© SPSS© Statistics version 23 (IBM© Corp., Armonk, NY). Continuous numerical variables are presented as mean and SD and categorical variables as numbers and percentages or ratios.

Inter-rater agreement was examined using the intraclass correlation coefficient (ICC). We used a two-way random model for a single measurement to calculate absolute agreement. The ICC is interpreted as follows: ICC values less than 0.5 are indicative of poor agreement, values between 0.5 and 0.75 indicate moderate agreement, values between 0.75 and 0.9 indicate good agreement, and values greater than 0.90 indicate excellent agreement according to Koo and Li [10]

Diagnostic accuracy for RSNA and CO-RADS classification was calculated for each Rater using RT-PCR as the gold standard for diagnosis. The following diagnostic indices were calculated: sensitivity, specificity, positive and negative predictive values, positive and negative likelihood ratios, and correct classification and misclassification rates.

## Results

This was a retrospective study conducted on 251 patients who underwent non-contrast CT and RT-PCR. One hundred and thirty-five patients presented with symptoms of suspicion for COVID-19 infection, while the other 116 patients came for hospital admission or preoperative and performed the RT-PCR as well as the CT as screening to avoid the spread of infection inside the non-isolated areas inside the hospital.

### Demographic data

**Fig. 1:**
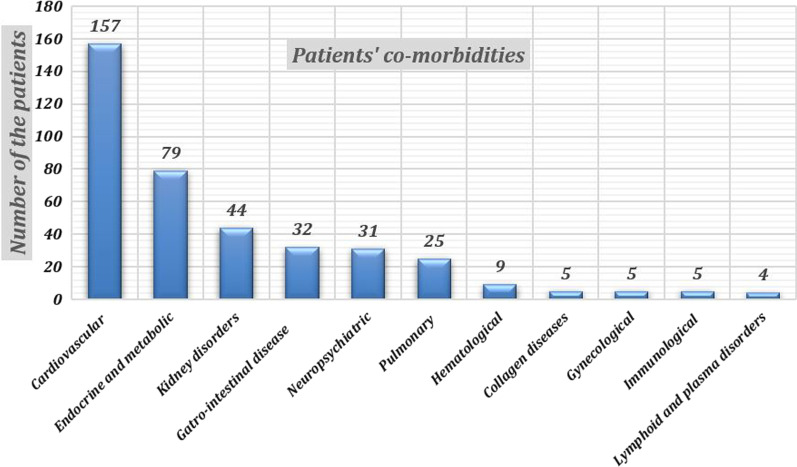
Illustrating the incidence of different comorbidities between our study population

The mean age of the patients was 52 ± 19 years ranging from 5 to 98 years. One hundred and thirty-seven patients were males representing 54.6%. Regarding the patients’ comorbidities, cardiovascular diseases were the most common (157/251) (62.5%) followed by DM (79/251) (31.5%) (Fig. 1).

The number of patients with positive RT-PCR for COVID-19 was 136 (54.2%) patients. The proportion of patients with positive RT-PCR for COVID-19 in typical, indeterminate, atypical, and negative categories according to the RSNA classification system ranged from 73.6–90.4%, 46.7–50.9%, 22.9–34%, and 38.2–48%, respectively. The proportion of patients with positive RT-PCR for COVID-19 in CORADS 1, 2, 3, 4, and 5 categories ranged from 40–48%, 25.6–30.6%, 42.9–45.2%, 50–79.2%, and 73.6–91.1%, respectively (Table 1).

**Table 1:** Number of patients assigned to each category in both classifications and percentage of confirmed RT-PCR COVID-19 cases

		PCR		Test value	*P*-value	Sig.
		Negative	Positive			
		No. = 115	No. = 136			
RSNA probability rater 1	Typical	7 (9.6%)	66 (90.4%)	56.221	0.000	HS
	Indeterminate	26 (53.1%)	23 (46.9%)			
	Atypical	35 (66.0%)	18 (34.0%)			
	Negative	47 (61.8%)	29 (38.2%)			
RSNA probability rater 2	Typical	23 (26.4%)	64 (73.6%)	23.947	0.000	HS
	Indeterminate	32 (53.3%)	28 (46.7%)			
	Atypical	21 (72.4%)	8 (27.6%)			
	Negative	39 (52.0%)	36 (48.0%)			
RSNA probability rater 3	Typical	15 (18.8%)	65 (81.2%)	44.139	0.000	HS
	Indeterminate	27 (49.1%)	28 (50.9%)			
	Atypical	37 (77.1%)	11 (22.9%)			
	Negative	36 (52.9%)	32 (47.1%)			
CO-RADS rater 1	1	48 (60.0%)	32 (40.0%)	55.532	0.000	HS
	2	34 (69.4%)	15 (30.6%)			
	3	23 (54.8%)	19 (45.2%)			
	4	5 (20.8%)	19 (79.2%)			
	5	5 (8.9%)	51 (91.1%)			
CO-RADS rater 2	1	39 (52.0%)	36 (48.0%)	24.254	0.000	HS
	2	21 (72.4%)	8 (27.6%)			
	3	16 (57.1%)	12 (42.9%)			
	4	16 (50.0%)	16 (50.0%)			
	5	23 (26.4%)	64 (73.6%)			
CO-RADS rater 3	1	39 (54.9%)	32 (45.1%)	34.104	0.000	HS
	2	32 (74.4%)	11 (25.6%)			
	3	11 (55.0%)	9 (45.0%)			
	4	12 (40.0%)	18 (60.0%)			
	5	21 (24.1%)	66 (75.9%)			

Data are expressed in number and percentage. The probability of error (*P*-value) at 0.05 was considered significant (*S*), 0.01 and 0.001 are highly significant (HS), while > 0.05 are considered non-significant (NS)

### Regarding the RSNA reporting system

**Fig. 2:**
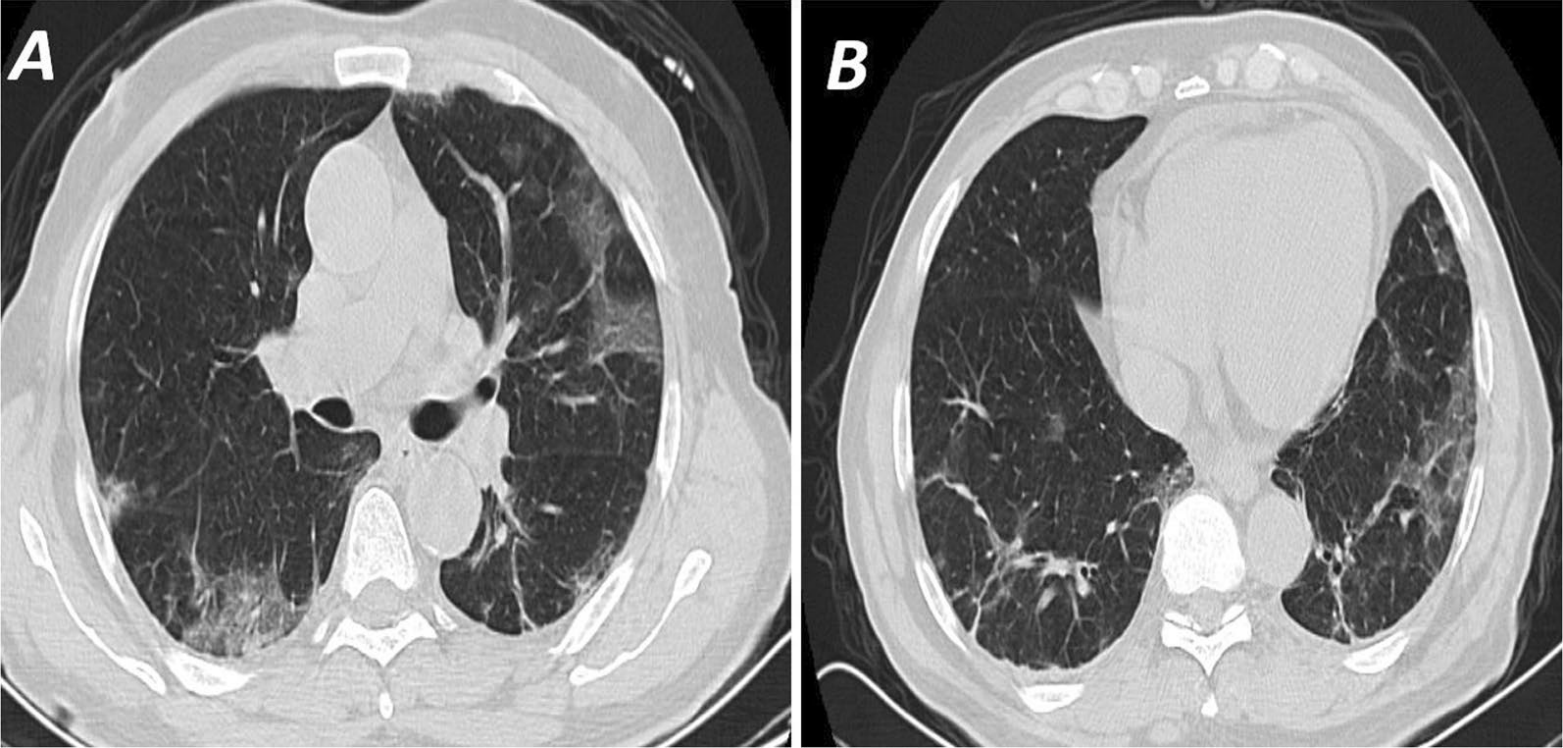
**A** and **B** High-resolution CT scan (axial cuts) for a 60 year-old diabetic male patient presented with fever and cough. CT shows multiple bilateral peripheral patches of ground-glass opacities in both lung lobes with linear consolidations seen in both lower lung lobes interpreted by all readers as a high probability (CO-RADS 5). RT-PCR was positive for COVID-19 and the patient responded to treatment and was discharged 14 days later

**Fig. 3:**
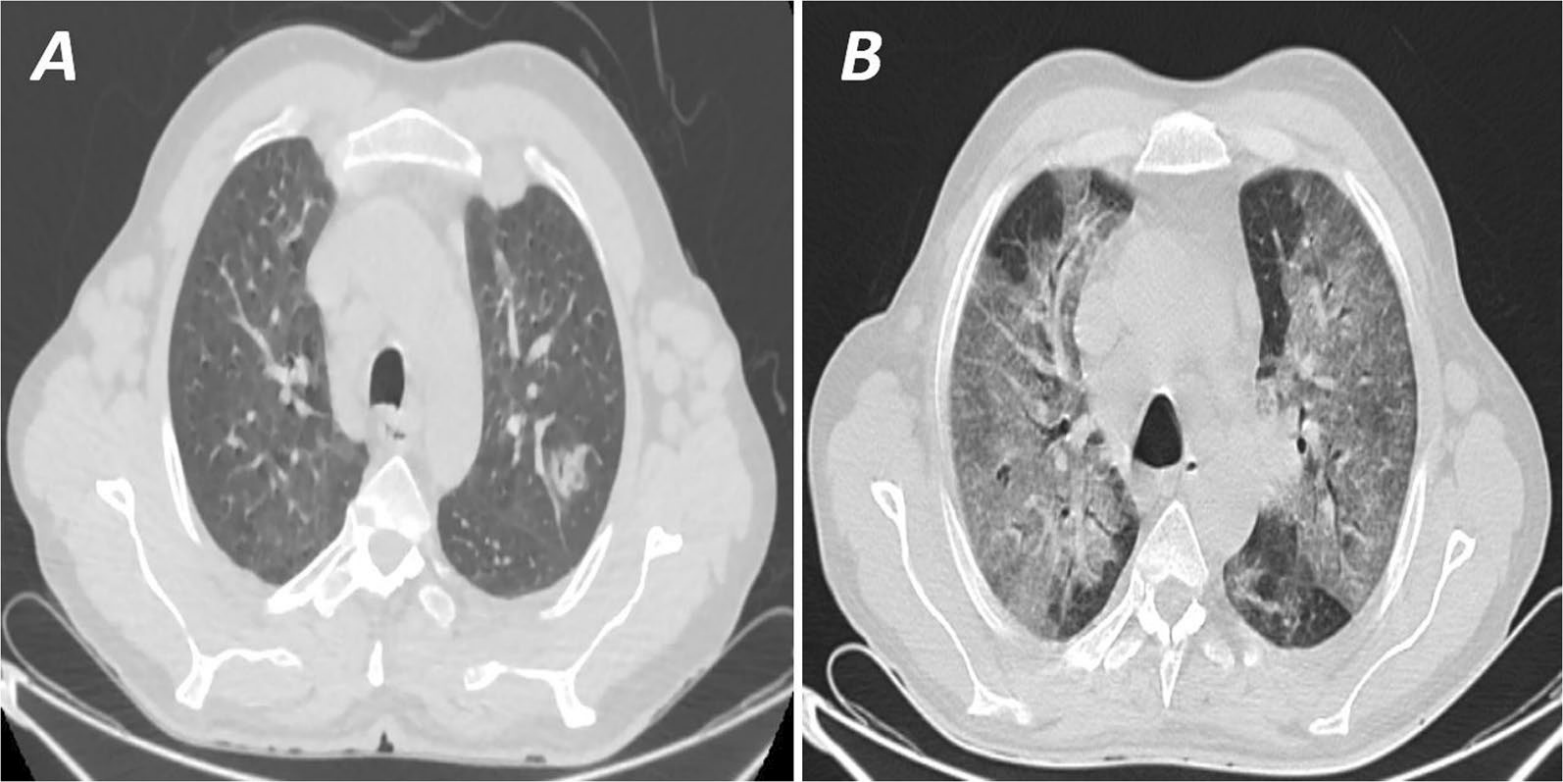
**A** High-resolution CT scan (axial cuts) for a 55-year-old male patient with recently diagnosed chronic myeloid leukemia presented with fever. The initial CT was interpreted as atypical for COVID (CO-RADS 2) with two patches of subsegmental consolidation in the left lobe and lingual. RT-PCR was positive for COVID-19.** B** CT scan was done nine days later after the patient developed desaturation shows a picture of non-cardiogenic pulmonary edema (ARDS), and the patient died a few days later

**Fig. 4:**
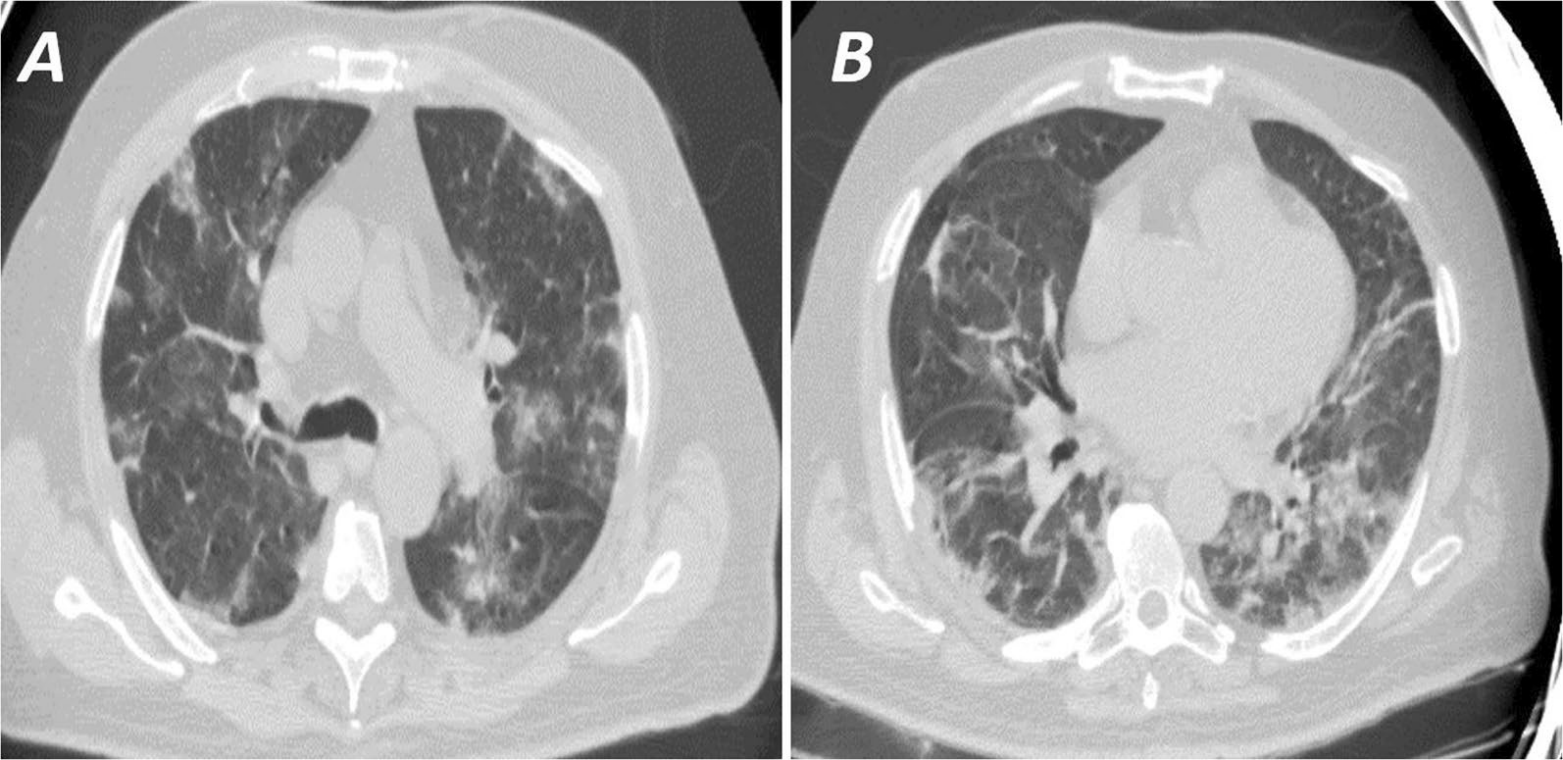
**A** and **B** High-resolution CT chest (axial cuts) for a 74-year-old diabetic hypertensive male patient presented with fever, cough, and dyspnea. CT was interpreted as typical (CO-RADS 5) by all readers. RT-PCR for COVID-19 was negative twice. The patient recovered from antibiotic treatment and was discharged a few days later

**Fig. 5:**
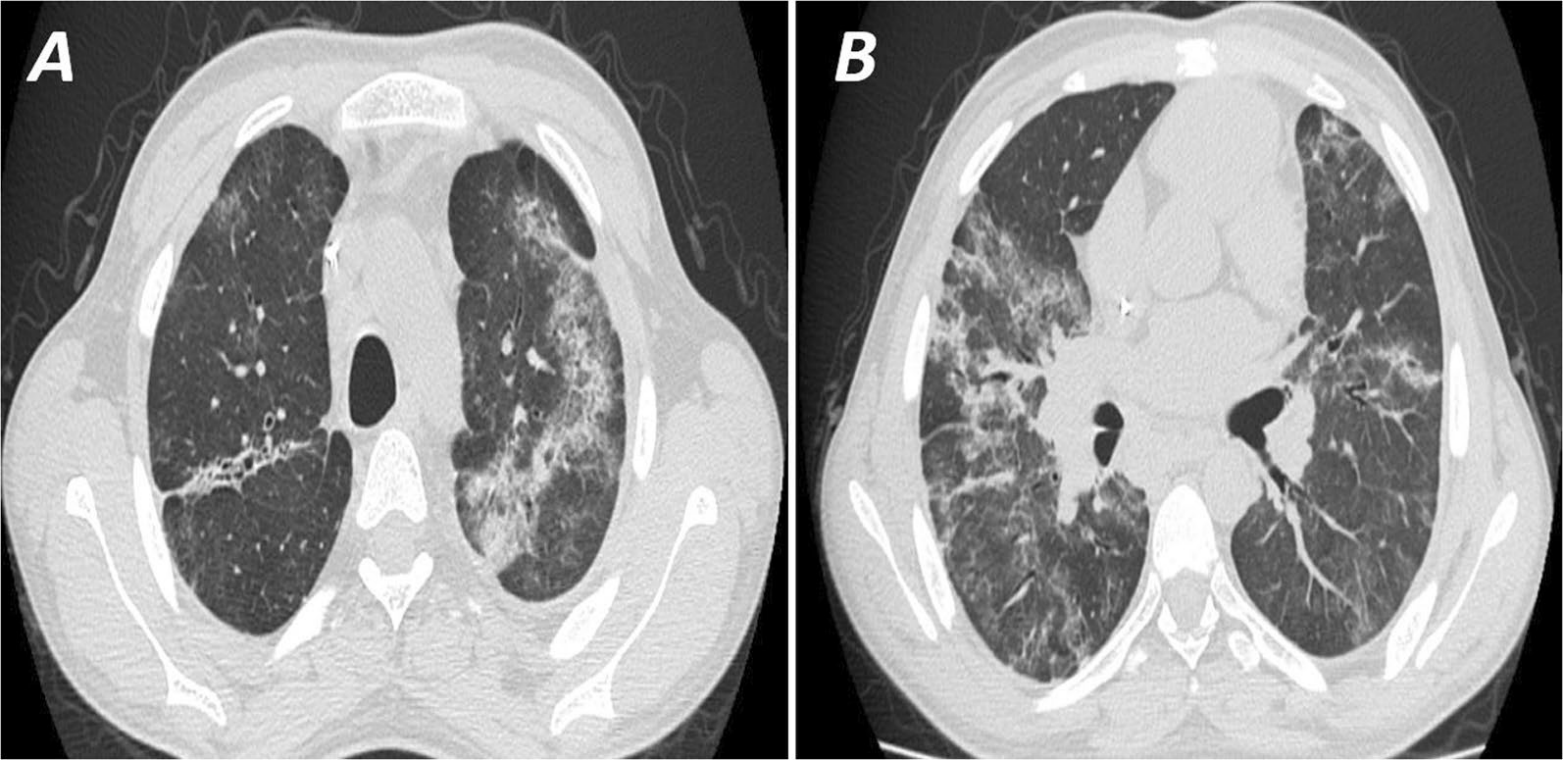
**A** and** B** High-resolution CT chest (axial cuts) for a 25-year-old male patient with common variable immune deficiency presented with fever, cough, and dyspnea. CT was interpreted as typical (CO-RADS 5) by all readers. RT-PCR for COVID-19 was positive. The patient improved and was discharged in two weeks

The sensitivity and specificity of the CT using the typical criteria established by RSNA recommendations ranged from 47–49% to 80–94%, respectively, for the three raters. The sensitivity and specificity of the typical and indeterminate categories together ranged from 65–68% to 52–71%, respectively, for the three raters. The positive predictive value while using the typical criteria ranged from 81 to 90%, while the negative predictive value ranged from 58 to 61%. The positive predictive value for typical and indeterminate groups ranged from 63 to 73%, while the negative predictive value ranged from 58 to 64%. (Table 2)*.* Reporting using the RSNA reporting system showed good inter-rater agreement with ICC = 0.77 (Table 3) (Figs. 2, 3, 4, 5).

**Table 2:** Accuracy of RSNA different classifications for diagnosis of COVID-19 as calculated for each of the three raters

	Rater 1			Rater 2			Rater 3		
	95% CI			95% CI			95% CI		
Statistic	RSNA atypical to typical	RSNA indeterminate to typical	RSNA typical	RSNA atypical to typical	RSNA indeterminate to typical	RSNA typical	RSNA atypical to typical	RSNA indeterminate to typical	RSNA typical
Correct classification	61%	68%	69%	55%	61%	62%	56%	66%	66%
Misclassification	39%	32%	31%	45%	39%	38%	44%	34%	34%
Sensitivity	79%	65%	49%	74%	68%	47%	76%	68%	48%
Specificity	41%	71%	94%	34%	52%	80%	31%	63%	87%
FPR	59%	29%	6%	66%	48%	20%	69%	37%	13%
FNR	21%	35%	51%	26%	32%	53%	24%	32%	52%
Prevalence	54%	54%	54%	54%	54%	54%	54%	54%	54%
PPV	61%	73%	90%	57%	63%	74%	57%	69%	81%
NPV	62%	64%	61%	52%	58%	56%	53%	63%	58%
Positive likelihood ratio	1.33	2.28	7.97	1.11	1.41	2.35	1.11	1.87	3.66
Negative likelihood ratio	0.52	0.48	0.55	0.78	0.62	0.66	0.75	0.50	0.60
Relative risk	1.60	2.00	2.30	1.18	1.48	1.68	1.21	1.86	1.96
Odds ratio	2.55	4.71	14.5	1.43	2.28	3.56	1.48	3.76	6.10

Data in cross-tables are counts.95% CI = 95% confidence interval.

*FPR* False-positive rate, *FNR* False-negative rate, *PPV* Positive predictive value, *NPV* Negative predictive value

**Table 3:** Inter-rater agreement for RSNA classification

		RSNA by rater 1				RSNA by rater 2			
		RSNA negative	RSNA atypical	RSNA indeterminate	RSNA typical	RSNA negative	RSNA atypical	RSNA indeterminate	RSNA typical
RSNA by rater 2	RSNA negative	63	7	3	2				
	RSNA atypical	3	23	3	0				
	RSNA indeterminate	9	16	26	9				
	RSNA typical	1	7	17	62				
RSNA by rater 3	RSNA negative	54	7	6	1	54	2	10	2
	RSNA atypical	10	30	6	2	7	22	16	3
	RSNA indeterminate	11	13	21	10	12	5	24	14
	RSNA typical	1	3	16	60	2	0	10	68

Inter-rater agreement	
Model, type	Two-way random, single measurement
Definition	Absolute agreement
Number of subjects (*n*), number of raters (*k*)	251, 3
Intra class correlation coefficient (ICC)	0.77*
95% CI	0.72–0.81

Data in cross-tables are counts. 95% CI = 95% confidence interval

*An ICC of 0.77 denotes good inter-rater reliability for RSNA classification

### Regarding the CO-RADS reporting system

The sensitivity and specificity of the CO-RADS 5 category ranged from 38–49% to 80–96% respectively for the three raters. The sensitivity and specificity of CO-RADS 4 and 5 categories together ranged from 51–62% to 66–91% respectively for the three raters. The sensitivity and specificity of CO-RADS 3, 4, and 5 categories together ranged from 65–68% to 52–71% respectively for the three raters. The positive predictive value for CO-RADS 5 group ranged from 74 to 91%, while the negative predictive value ranged from 56 to 57%. The positive predictive value for CO-RADS 4 and 5 groups ranged from 72 to 88%, while the negative predictive value ranged from 58 to 61%. The positive predictive value for CO-RADS 3, 4 and 5 groups ranged from 63 to 73%, while the negative predictive value ranged from 58 to 64%. (Table 4). Reporting using the CO-RADS reporting system showed good inter-rater agreement with ICC = 0.75 which was close and similar to the RSNA reporting system (Table 5) (Figs. 2, 3, 4, 5).

**Table 4:** Accuracy of different CO-RADS classes for diagnosis of COVID-19 as calculated for each of the three raters

	Rater 1				Rater 2				Rater 3			
	95% CI				95% CI				95% CI			
Statistic	CO-RADS 2–5	CO-RADS 3–5	CO-RADS 4–5	CO-RADS 5	CO-RADS 2–5	CO-RADS 3–5	CO-RADS 4–5	CO-RADS 5	CO-RADS 2–5	CO-RADS 3–5	CO-RADS 4–5	CO-RADS 5
Correct classification	61%	68%	70%	64%	55%	61%	62%	62%	57%	65%	66%	64%
Misclassification	39%	32%	30%	36%	45%	39%	38%	38%	43%	35%	34%	36%
Sensitivity	76%	65%	51%	38%	74%	68%	59%	47%	76%	68%	62%	49%
Specificity	42%	71%	91%	96%	34%	52%	66%	80%	34%	62%	71%	82%
FPR	58%	29%	9%	4%	66%	48%	34%	20%	66%	38%	29%	18%
FNR	24%	35%	49%	63%	26%	32%	41%	53%	24%	32%	38%	51%
Prevalence	54%	54%	54%	54%	54%	54%	54%	54%	54%	54%	54%	54%
PPV	61%	73%	88%	91%	57%	63%	67%	74%	58%	68%	72%	76%
NPV	60%	64%	61%	56%	52%	58%	58%	56%	55%	62%	61%	57%
Positive likelihood ratio	1.31	2.28	5.92	8.63	1.11	1.41	1.73	2.35	1.16	1.79	2.15	2.66
Negative likelihood ratio	0.56	0.48	0.53	0.65	0.78	0.62	0.62	0.66	0.69	0.51	0.54	0.63
Relative risk	1.52	2.00	2.27	2.09	1.18	1.48	1.58	1.68	1.28	1.80	1.85	1.78
Odds ratio	2.33	4.71	11.14	13.20	1.43	2.28	2.78	3.56	1.67	3.49	4.01	4.22

Data in cross-tables are counts.95% CI = 95% confidence interval.

*FPR* False-positive rate, *FNR* False-negative rate, *PPV* Positive predictive value, *NPV* Negative predictive value

**Table 5:** Inter-rater agreement for CO-RADS classification

		CO-RADS by rater 1					CO-RADS by rater 2				
		CO-RADS 1	CO-RADS 2	CO-RADS 3	CO-RADS 4	CO-RADS 5	CO-RADS 1	CO-RADS 2	CO-RADS 3	CO-RADS 4	CO-RADS 5
CO-RADS by rater 2	CO-RADS 1	64	6	2	2	1					
	CO-RADS 2	4	22	3	0	0					
	CO-RADS 3	6	10	8	2	2					
	CO-RADS 4	4	5	16	6	1					
	CO-RADS 5	2	6	13	14	52					
CO-RADS by rater 3	CO-RADS 1	55	8	7	0	1	55	3	10	1	2
	CO-RADS 2	10	27	3	3	0	6	21	7	6	3
	CO-RADS 3	5	6	6	3	0	5	3	4	4	4
	CO-RADS 4	8	4	8	7	3	7	2	3	12	6
	CO-RADS 5	2	4	18	11	52	2	0	4	9	72

Inter-rater agreement	
Model, type	Two-way random, single measurement
Definition	Absolute agreement
Number of subjects (*n*), number of raters (*k*)	251, 3
Intra class correlation coefficient (ICC)	0.75*
95% CI	0.70–0.80

Data in cross-tables are counts. 95% CI = 95% confidence interval

*An ICC of 0.75 denotes good inter-rater reliability for CO-RADS classification

The diagnostic accuracy of the two reporting systems, as well as the inter-rater agreement, were almost similar (Tables 2 and 4).

### Regarding the use of CT in screening asymptomatic patients

For simplicity and with the base of good inter-rater agreement found between the three raters in the current study, we used the reading of rater 1 who is the more experienced in the analysis of the results among symptomatic and asymptomatic cases. Among 43 asymptomatic cases with positive RT-PCR test for COVID-19 infection, the CT detected changes of high to indeterminate probability for COVID-19 infection in only 17 cases (39.5%). While in 93 symptomatic cases with positive RT-PCR, the CT detected changes of high to indeterminate probability for COVID-19 infection in 72 cases (77.4%). The CT showed a highly significant *P*-value of 0.000 when used among clinically suspected patients in controversy to a non-significant *P*-value of 0.95/0.96 when used as screening in asymptomatic patients (Table 6). Negative CT and indeterminate probability were the highest interpretation detected among asymptomatic patients during the use of the RSNA reporting system, while in the CO-RADS reporting system, CO-RADS 1 and 3 were the commonest.

**Table 6:** Number of patients assigned to each category in both classifications according to presence or absence of symptoms as well as the confirmed RT-PCR

	Symptomatic patients		*P*-value	Asymptomatic patients		*P*-value
	PCR + ve	PCR − ve		PCR + ve	PCR − ve	
*RSNA reporting system*						
Typical	60	5	0.000 (HS)	6	2	0.95 (NS)
Indeterminate	12	7		11	19	
Atypical	11	20		7	15	
Negative	10	10		19	37	
Total	93	42		43	73	251
	135			116		251
*CO-RADS reporting system*						
CO-RADS 1	11	9	0.000 (HS)	21	39	0.96 (NS)
CO-RADS 2	10	20		5	14	
CO-RADS 3	9	6		10	17	
CO-RADS 4	16	3		3	2	
CO-RADS 5	47	4		4	1	
Total	93	42		43	73	251
	135			116		251

Data are expressed in number. The probability of error (*P*-value) at 0.05 was considered significant (*S*), 0.01 and 0.001 are highly significant (HS), while > 0.05 are considered non-significant (NS)

### Discussion

In this study, the authors tried to evaluate the inter-observer agreement while reporting the CT using RSNA and CO-RADS reporting systems and showed good inter-observer agreement even with the difference in the radiologists’ experiences. Also, almost similar diagnostic accuracy was found while using the two reporting systems. CT showed non-significant values in the detection of COVID-19 infection while used in the screening of asymptomatic patients.

The American College of Radiology and the Royal College of Radiologists recommended that CT should not be used to screen patients with suspected COVID-19 infection [11, 12]. However, chest CT was used in our hospital as a method of triaging patients with suspected COVID-19 infection, as well as to exclude infection in patients considered for hospitalization for other reasons, especially with the scarcity and time-consuming nature of the RT-PCR test and if there was any clinical suspicion of COVID-19 infection [13]. The RSNA chest CT classification for reporting COVID 19 pneumonia provides a method for conveying to the clinicians the level of suspicion of COVID-19 [7]. In March 2020, the Dutch Radiological Society developed the CO-RADS reporting system to classify pulmonary involvement by COVID-19 [5].

In this study, we found the proportion of patients with RT-PCR confirmed COVID-19 was highest in the typical followed by the indeterminate category of the RSNA system. The proportion of RT-PCR confirmed cases in the negative category (38.2–48%) was higher than that in the atypical category (22.9–34%). This is concurrent with De Jaegere et al. [8] who explained this by the higher prevalence of other lung diseases in the atypical category (e.g., bacterial pneumonia and pulmonary edema) compared to the negative category, which was also the case in our study. Regarding the CO-RADS system, similar proportions were reported for RT-PCR confirmed cases with the CO-RADS 1 category showing a higher proportion of positive cases (40–48%) than the CO-RADS 2 category (25.6–30.6%). This was also in agreement with De Jaegere et al. [8]

Reporting using the RSNA reporting system showed good inter-rater agreement with ICC = 0.77. This is slightly lower than Byrne et al. [14] who reported substantial agreement in the indeterminate category and almost perfect agreement in the other three categories. De Jaegere et al. [8] reported substantial to a moderate overall agreement**.** An Italian study by Ciccarese et al. [15] reported a moderate interobserver agreement**.**

We found that reporting using the CO-RADS reporting system showed good inter-rater agreement with ICC = 0.75 which is slightly higher than Prokop et al. [5] who reported moderate reliability with a *k* value of 0.47. Fujioka et al. [16] reported substantial to an almost perfect interobserver agreement in the CO-RADS system with ICC 0.800–0.874 which is considered slightly higher than that in our study.

The sensitivity of the typical category in the RSNA reporting system ranged from 47 to 49% for the three readers. When using the indeterminate category as the threshold, the sensitivity for typical and indeterminate categories increased to 65–68%. The sensitivity in our study is remarkably less than that reported by Som et al. [17] who reported a sensitivity of typical and indeterminate findings to be 97.5% (range 94–100%)**.** This could likely be explained by the use of CT in our hospital for symptomatic patients suspected to be infected by COVID-19 as well as a screening tool for patients considered for hospitalization for other reasons, while in their study, CT was used as a problem-solving tool in suspected cases and to guide management, which implied that all patients were symptomatic at the time of imaging, which was not the case in our study. Our findings concur with Kim et al. [18] who reported that CT may be normal in asymptomatic and early cases of COVID-19 infection**.** A meta-analysis done by Kwee et al. [19] reported a pooled sensitivity of 65.2% for the typical category and 90.2% for typical and indeterminate categories**,** also higher than the values in the current study.

The sensitivity of the CO-RADS 5 category ranged from 38 to 49%. When using CO-RADS 4 and 5 categories as a threshold, the sensitivity ranged from 51 to 62%. The sensitivity of CO-RADS 3, 4, and 5 categories together increased to 65–68% in our study. Our values were lower than those by Kwee et al. [19] who reported a pooled sensitivity of CO-RADS-5 category 70.4%, for CO-RADS 4 and 5 categories increased to 85.8%, and for CO-RADS 3, 4, and 5 categories were reaching 92.5%**.**

Regarding the specificity of the typical category in our study, it ranged from 80–94% and decreased to 52–71% for the typical and indeterminate categories, concurrent with Som et al. [17] who reported a specificity of 54.7% for the latter categories**.** The high specificity of the typical category was higher than that reported in a large study in China by Ai et al. [20], who reported a specificity of 25% for chest CT. This was explained in their study by the possibility of RT-PCR being falsely negative in many cases. In these patients, they performed a further analysis based on exposure history, clinical manifestations, and serial CT scans, to better classify patients with positive CT findings and negative RT-PCR tests**.** Similarly, we reported the specificity of CO-RADS 5 category to range from 80 to 96%, CO-RADS 4 and 5 ranged from 66 to 91% and decreased to 52–71% for CO-RADS 3, 4 and 5 categories, concurrent with the literature stating that at high diagnostic thresholds, specificity increases at the cost of sensitivity [19]

Finally, our results concerning the non-significant value of CT while used in the screening of asymptomatic patients are consistent with the ACR recommendations [11, 12]. Smet et al. [21] found that CO-RADS 3 was the most commonest among positive asymptomatic cases and concluded CT screening for asymptomatic patients is not recommended which is similar to the current study.

## Limitations

Our study had several limitations. First the use of RT-PCR as a gold standard for COVID-19 infection with its reported false-negative rate that could reach 63% in nasopharyngeal swabs [22]. Second, RT-PCR was not repeated in all negative cases, yet in patients with persistent clinical suspicion of COVID-19, at least two RT-PCR tests were done. The study was also limited by its retrospective design and the lack of clinical data regarding the duration of symptoms at the time of CT scanning. Among the strengths of the study was the inclusion of symptomatic and asymptomatic cases rendering it more valuable in the assessment of the diagnostic performance of CT without selection bias.

## Conclusion

There is a good inter-rater agreement while reporting using either the RSNA recommendations or the CO-RADS reporting systems among radiologists with different levels of experience, rendering them useful in providing standardized reports to convey the level of suspicion of COVID-19 infection to the clinicians. Despite the high specificity of CT in the diagnosis of symptomatic COVID-19, especially with high diagnostic thresholds, our study concluded that its sensitivity is lacking owing to the considerable number of confirmed COVID-19 cases in the negative and atypical categories, limiting its use as a screening test in asymptomatic cases in controversy to what happened while used in symptomatic patients. So, it is not suggested to use CT as screening for COVID-19 infection in asymptomatic patients.

## Data Availability

The datasets used and/or analyzed during the current study are available from the corresponding author on reasonable request.

## Abbreviations

COVID-19: Corona Virus Disease 2019
CT: Computed tomography
RT-PCR: Reverse transcription-polymerase chain reaction
RSNA: Radiological society of North America
CO-RADS: Coronavirus disease reporting and data system
